# Mixed methods evaluation of the impact of the COVID-19 ICU remote-learning rehabilitation course for frontline health professionals during the COVID-19 pandemic in the UK

**DOI:** 10.1177/17511437211043043

**Published:** 2021-09-23

**Authors:** Evelyn J Corner, Xiaoxi Zhang, Zoe Van Willigen, Kate Tatam, Matthew Camilleri, Alex Monkhouse, Danielle E Bear, Alex Hemsley, Zudin Puthucheary, Alex Rosenberg, Jackie McRae, Alex Harvey, Debbie Ford, Penelope Firshman, Meriel Norris

**Affiliations:** 1 Department of Health Sciences, 3890 Brunel University London, Uxbridge, London, UK; 2 8946 Imperial College NHS Healthcare Trust, Fulham Palace Road, London, UK; 3 33N Ltd, London, UK; 4 Clinically-Led WorkforcE and Activity Redesign (CLEAR) Programme, Health Education England, London, UK; 5 Department of Anaesthesia, Hillingdon Hospital, London, UK; 6 Therapy Services Department, 7425 University Hospital Southampton NHS Foundation Trust, Southampton, UK; 7 Derriford Hospital, University Hospitals Plymouth NHS Trust, Plymouth, UK; 8 Anaesthetics Department, Peterborough City Hospital, Peterborough, UK; 9 Departments of Nutrition and Dietetics and Critical Care, 8945 Guy’s and St Thomas’ NHS Foundation Trust, London, UK; 10 Physiotherapy Department, Newcastle Upon Tyne Hospitals NHS Foundation Trust, Newcastle Upon Tyne, UK; 11 Anaesthetics Department, St Bartholomew’s Hospital, London, UK; 12 William Harvey Research Institute, Queen Mary University of London, London, UK; 13 Critical Care and Perioperative Medicine Research Group, 112001 The Royal London Hospital, London, UK; 14 Critical Care and Cardiothoracic Services, Royal Brompton and Harefield Hospitals, Guys and St Thomas’s NHS Trust, London, UK; 15 Adult Speech and Language Therapy Department, 8964 University College Hospitals NHS Foundation Trust, London, UK; 16 Staff Psychology, 4964 Royal Brompton and Harefield Hospitals, Guys and St Thomas’s NHS Trust, London, UK; 17 111990 King’s College Hospital NHS Foundation Trust, London, UK

**Keywords:** COVID-19, pandemics, education, critical care, rehabilitation

## Abstract

**Background:**

Optimising outcomes for critically ill patients with COVID-19 patients requires early interdisciplinary rehabilitation. As admission numbers soared through the pandemic, the redeployed workforce needed rapid, effective training to deliver these rehabilitation interventions.

**Methods:**

The COVID-19 ICU Remote-Learning Rehab Course (CIRLC-rehab) is a one-day interdisciplinary course developed after the success of CIRLC-acute. The aim of CIRLC-rehab was to rapidly train healthcare professionals to deliver physical, nutritional and psychological rehabilitation strategies in the ICU/acute setting. The course used blended learning with interactive tutorials delivered by shielding critical care professionals. CIRLC-rehab was evaluated through a mixed-methods approach, including questionnaires, and follow-up semi-structured interviews to evaluate perceived impact on clinical practice. Quantitative data are reported as *n* (%) and means (SD). Inductive descriptive thematic analysis with methodological triangulation was used to analyse the qualitative data from the questionnaires and interviews.

**Results:**

805 candidates completed CIRLC-rehab**.** 627 (78.8%) completed the post-course questionnaire. 95% (*n* = 596) found CIRLC-rehab *extremely* or *very* useful and 96.0% (*n* = 602) said they were *very likely* to recommend the course to colleagues. Overall confidence rose from 2.78/5 to 4.14/5. The course promoted holistic and humanised care, facilitated informal networks, promoted interdisciplinary working and equipped the candidates with practical rehabilitation strategies that they implemented into clinical practice.

**Conclusion:**

This pragmatic solution to educating redeployed staff during a pandemic increased candidates’ confidence in the rehabilitation of critically ill patients. There was also evidence of modifications to clinical care utilising learning from the course that subjectively facilitated holistic and humanised rehabilitation, combined with the importance of recognising the humanity, of those working in ICU settings themselves. Whilst these data are self-reported, we believe that this work demonstrates the real-term benefits of remote, scalable and rapid educational delivery.

## Introduction

To date, over 30,000 patients have been admitted to intensive care units (ICU) with COVID-19 since March 2020.^1^ ICU survivors can suffer from a range of physical, cognitive and psychological sequalae that can affect their recovery for months or years after ICU discharge.^2^ This includes, but is not limited to, muscle wasting and weakness, reduced exercise tolerance, dyspnoea, speech and swallowing difficulty, nutritional deficit, post-traumatic stress disorder, anxiety and depression.^3^ COVID-19 patients may experience prolonged immobility due to ventilatory support with deep sedation, which can exacerbate these complications.^4^ This can lead to a reduced health-related quality of life and increased healthcare service utilisation.

Pre-pandemic, early rehabilitation of ICU patients has proved safe and feasible^5^ and is recommended by NICE (2010) to attenuate some of the complications associated with an ICU stay.^6^ Recent data from the National Post-Intensive Care Rehabilitation Collaborative showed that COVID-19 patients required input from all possible rehabilitation disciplines during their stay. In addition, 13% of COVID-19 survivors required inpatient rehabilitation after hospital discharge, and 36% required community rehabilitation, demonstrating the need to deliver early interdisciplinary rehabilitation strategies in this cohort.^7,8^

However, safe staffing levels are important to implement early rehabilitation effectively. During the COVID-19 pandemic in the United Kingdom, the patient-to-staff ratio increased 13-fold for psychological services and threefold for most other disciplines (ICS), highlighting huge gaps in the critical care workforce.^9^ This has mandated rapid education and cross-skilling of non–critical care trained staff to deliver both acute care and early rehabilitation in the acute setting. With clinical staff and services stretched to their limit, training redeployed staff to deliver this care created an additional work burden. At the same time, many highly skilled critical care clinicians were forced to ‘shield’ at home due to medical risk factors.

This article describes the mixed-methods evaluation of the COVID-19 ICU Remote-Learning Rehabilitation Course (CIRLC-rehab), a remote interdisciplinary training course in ICU rehabilitation. This is the second course that we have developed in response to the COVID-19 pandemic. The first course (COVID-19 ICU Remote Learning Course (CIRLC)) focused on the acute care of the critically ill patient.^10^ The principle underpinning both courses was to ensure consistent delivery of education in a social-distanced manner using appropriate technology. The courses consisted of pre-recorded lectures followed by interactive tutorials delivered by shielded, experienced ICU clinicians. This would offload the teaching workload of those able to work on the frontline, freeing them up for patients facing care whilst giving shielding clinicians a valuable role in the pandemic. The development, underpinning educational theory, implementation and results of the CIRLC-acute course was published elsewhere.^10^ As the CIRLC-rehab course used a similar process of iterative development, this current article will focus more on the mixed-methods evaluation of the course: in particular, the perceived impact of the course on clinical practice through follow-up semi-structured interviews.

## Methods

Key experts in the field of rehabilitation in critical illness were approached from multi-professional backgrounds: nursing (KT), physiotherapy (ZVW, AH and EJC), speech and language therapy (JM), dietetics (DB), medicine (AR and ZP), occupational therapy (PF) and psychology (DF). An online working group was pulled together to decide on the content and format for the course. Session plans and learning objectives for each topic were developed (see Table 1).

**Table 1.** COVID ICU Rehabilitation Remote-Learning Course learning objectives.

Opening session: A day in the life of an ICU patient with COVID-19	Have an understanding of the lived experience of an ICU survivor
	Have a basic grasp of day-to-day life for a patient in ICU
Weaning from mechanical ventilation and tracheostomy	Know how to establish when a patient is ready to wean from mechanical ventilation
	Understand the weaning process including tracheostomy weaning
	Be able to identify failure to wean and act accordingly
	Understand the impact of a tracheostomy on speech, swallow and secretion management
Intensive care unit–acquired weakness (ICUAW)	Have an understanding of the pathophysiology of intensive care unit–acquired weakness (ICUAW)
	Be able to clinically diagnose and assess ICUAW
Early mobilisation and physical rehabilitation of the ICU patient	Know when it is safe to start early mobilisation in the ICU
	Have an understanding of early mobilisation strategies
	Understand COVID-19–specific rehabilitation issues, for example, breathlessness and pacing.
Nutritional issues in critical illness	Understand the impact of critical illness on the patient’s nutritional status
	Understand how to screen for nutritional risk in ICU patients
	Understand how to support and document nutritional intake
Delirium	Know what delirium is and how to assess it
	Have an understanding of delirium management strategies
	Understand the importance of humanised care
The psychological impact of critical illness	Understand the psychological impact of critical illness
	Be able to implement some simple psychological strategies
	To be able to identify those at risk of psychological morbidity
	To consider psychological issues in palliative care
Closing session – goal setting and rehab planning	Be able to formulate a rehabilitation plan for my patient
	Be able to identify appropriate goals in relation to their rehabilitation
	To consider simple strategies to support patients in the community

Following the successful application of the ‘flipped classroom’ model using blended learning during CIRLC-acute, the same approach was adopted for CIRLC-rehab.^10^ Students were able to watch pre-recorded material at their own pace. This was followed by expert-led group tutorials to consolidate key concepts of the lectures. The small-group interactive sessions enabled tutors to tailor their teaching to accommodate different learning styles. Furthermore, tutors and students were encouraged to share their own experience of caring for COVID-19 patients to enhance learning. A key difference between the CIRLC-acute course ^10^ and CIRLC-rehab is that CIRLC-rehab was delivered using inter-professional learning. This was chosen because rehabilitation in critical illness requires an inter-professional approach.^11^

## Methods of evaluation

The programme was primarily evaluated through an online survey administered before and immediately after the course. Its development was informed by Sitzman & Weinhardt^12^ and included demographic information of attendees (to assess training utilisation), Likert-based questions regarding confidence of knowledge (training affect) and open questions regarding participants’ experience of the course. Performance indicators could not be directly assessed due to the nature of the pandemic, but all attendees were invited to participate in an online or telephone semi-structured interview once back in practice, in which topics included motivation for course attendance, reflection on course content and critically potential impact of the course on practice. Appropriate ethical approval was gained for the evaluation phase from Brunel University Research Ethics Committee, and all data have been anonymised. The interviews were undertaken by an experienced qualitative researcher independent of the delivery of the course, a point the participants were aware of to encourage honest reflections. Interviews were audio-recorded and transcribed verbatim.

## Data analysis

The questionnaire data were predominantly analysed using descriptive statistics. Open comments from the questionnaire and interview data were independently analysed by two researchers (EC and MN) through inductive descriptive thematic analysis.^13^ This atheoretical approach was deemed suitable for a pragmatic evaluation. Steps included familiarisation of the data, line coding and collation of subthemes and themes. Following the development of the themes, the two qualitative datasets were integrated through methodological triangulation. In this process, the independent themes were inputted into a matrix and reviewed for convergence/agreement, complementarity/partial agreement, and dissonance and silence with the overall development and reporting of integrated themes.^14,15^ This final analytical stage was completed collaboratively by two researchers (MN and EC).

## Results

CIRLC-rehab ran 16 times during from May 2020–July 2020, training 692 candidates. CIRLC-rehab restarted in November 2020 through January 2021, running a further 10 times and training 113 candidates. This gives a total of 805 candidates. Between 5 and 48 candidates attended the course/day.

## Demographics

Most candidates were physiotherapists (62.6%) followed by occupational therapists (14.8%), from the specialities of medicine (35.8%), intensive care and anaesthetics (20.7%) or rehabilitation medicine (12.9%). 37 different specialities were represented.

The majority of candidates were Agenda for Change pay bands 5 (28.2%), 6 (38.3%) and 7 (21.7%). 18% had no ICU experience. Full demographics of course candidates can be found in Table 2.

**Table 2.** Candidate demographics.

Profession	Number	%
Physiotherapist	499	62.6
Occupational therapist	118	14.8
Dietitian	30	3.8
Speech and language therapist	29	3.6
Rehabilitation assistant	29	3.6
Doctor	23	2.9
Nurse	63	7.9
Other	5	0.6
Area of practice		
Medicine	285	35.8
Surgery	68	8.5
Trauma and orthopaedics	25	3.1
Intensive care medicine and anaesthetics	165	20.7
General practice	13	1.6
Infectious diseases	12	1.5
Paediatrics	20	2.5
Oncology	9	1.1
Neurology/neurosurgery	74	9.3
Emergency medicine	13	1.6
Rehabilitation medicine	103	12.9
Other^a^	9	1.1
Clinical grade		
Band 1–4	49	6.2
Band 5	225	28.2
Band 6	305	38.3
Band 7	173	21.7
Band 8	22	2.8
Consultant	9	1.1
Specialist trainee 1–5/fellow	13	1.6
Prior experience in ICU/HDU		
Nil	142	18.0
< 6 months	259	32.5
6–24 months	210	26.4
> 24 months	185	23.2
Last worked in ICU/HDU		
Never	140	17.6
< 1 year ago	532	66.8
1–2 years ago	44	5.5
3–5 years ago	24	3.0
> 5 years ago	56	7.0

^a^other included: occupational medicine, maxillofacial surgery, plastic surgery, public health medicine, vascular, sports medicine, renal, palliative care, psychiatry, gastroenterology and public health.

## Pre-post questionnaire results

Of the 805 attendees, 796 (98.8%) candidates completed the pre-course questionnaire, and 627 (78.8%) completed the post-course questionnaire. The results showed that overall confidence increased from a mean of 2.78/5 to 4.14/5; this was replicated in all topic areas with confidence around nutritional issues increasing the most from 2.29 to 3.87. Those with fewer years' experience in the ICU benefitted the most from the course. Table 3 summarises the key findings.

**Table 3.** Questionnaire results-changes in confidence levels by topic and experience.

	Before, group mean (SD)	After, group mean (SD)	Mean difference
Confidence increase in Likert scale by topic			
Weaning from mechanical ventilation and tracheostomy	2.49 (1.25)	3.91 (0.94)	1.42 increase
Intensive care unit–acquired weakness (ICUAW)	2.38 (1.09)	3.92 (0.77)	1.54 increase
Early mobilisation and physical rehabilitation of the ICU patient	2.95 (1.23)	4.36 (0.74)	1.41 increase
Nutritional issues in critical illness	2.29 (1.13)	3.87 (0.81)	1.58 increase
Delirium	2.98 (1.10)	4.25 (0.71)	1.27 increase
The psychological impact of critical illness	2.84 (1.03)	4.16 (0.71)	1.32 increase
Goal setting and rehab planning	3.53 (1.06)	4.53 (0.65)	1.00 increase
Likert scale confidence increase by prior ICU experience (all professions and topics)			
None	2.10 (1.18)	3.84 (0.91)	1.74
Less than 6 months	2.62 (1.14)	4.08 (0.78)	1.46
6 months to 2 years	3.00 (1.09)	4.24 (0.69)	1.24
More than 2 years	3.21 (1.15)	4.31 (0.81)	1.10

95% (*n* = 596) of candidates found CIRLC-rehab *extremely* or *very* useful and 4.9% (*n* = 31) rated it as a *little* useful. 96.0% (*n* = 602) said they were *very likely* and (*n* = 20) 3.6% said they were a *little likely* to recommend the course to colleagues.

## Qualitative results

Twelve participants volunteered and undertook telephone interviews (mean = 35 min, range = 23–51 min). They included 10 physiotherapists, a nurse and a speech and language therapist, which is representative of the candidate distribution. All but one was redeployed into COVID-19–related duties. They had been qualified for between 5 months and 25 years (mean = 8 years).

The analytical triangulation process resulted in the development of three major themes: A need to safely expand a holistic toolkit, re-humanising of patient and self, and changing practice and reconceptualising roles.

### A need to safely expand a holistic toolkit

Participants, predominantly from the interviews, noted that their practice has been based on previous rehabilitation experience, and there was a need for them to review this based on more specific COVID-19 expertise. Part of that related to an initial lack of confidence in their own practice, but also the potential to plug knowledge gaps:

“my knowledge of trache weaning is really limited…but even having the confidence to know what stage of the wean they are…knowing when to time their rehab…I wouldn’t have thought about it as much…having the full understanding and confidence that I know what this means and I know I can do this.” (Physio, 1 year qualified)

As indicated here, some of the gaps related to the stages of rehabilitation. Others included potential tools, such as patient diaries, activity charts or appropriate outcome measures that participants had been unaware of. They also noted the value of exploring the patient’s journey to assist in the understanding of their complex presentation. Through this, participants, while not always expecting it from the course, reflected on the benefits of being introduced and able to engage with the bigger picture. A consistent feature of this was the input from different members of the interdisciplinary team (IDT), further supported by discussions in the mixed-participant groups. Participants highlighted a range of new insights, but the focus on nutrition was frequently noted.

“I found the dietitians lecture very interesting because again, something we kind of skim over… but it massively affects what we do. You know, they don’t have the right nutrition and they don’t have the right energy levels. They want rehab…But it’s something we just kind of brush off and don’t really look into... I found that really interesting” (Physio, 3 years)

While the formal teaching itself was discussed very positively, the more informal and ‘safe’ tutorial format supported these insights. Approachable leaders, the ability to anonymously ‘chat’ and sharing of varied experiences were highly valued.

### Re-humanisation of care patients and self

During the pandemic, intensive care units were the busiest they have ever been, with experienced staff attempting to manage high volumes of critically ill patients whilst simultaneously supervising novice clinicians who were working at the bedside – all of this whilst wearing personal protective equipment (PPE). This created a highly stressful and morally distressing environment. Skill-based teams were also the norm, with proning teams ‘flipping’ patients over and over throughout the day. Participants reported that this was one of the hardest parts of working during the pandemic, creating a dehumanising environment for both the patients and the staff.

They commented how the course challenged that view by putting the patient at the centre of care. It helped them to remember and recognise that in the chaotic environment of a pandemic, with staff stress and personal protection being of grave concern, there was a human being at the centre of it all:

“The course really honed in on patient centred care. Physios do that anyway but with COVID it was so busy you were focused on everyone and not taking on in consideration what you would usually do with the patient themselves… it made me think we need to go back and think what do they want to actually do.... (it) made me realise that maybe we had been grouping everyone together a bit, and helped focus on patient centred care.” (Physio, 1 year)

While the re-focusing was particularly noted by more recently qualified staff, those with more experience drew on interactions with the psychologist to reconsider their own position:

“I mentioned something on the comment on the chat box about how we as staff members feel and how it impacts on those seeing patients in this way. You know, it’s so distressing to they’re very often they are the same age as our parents or relatives or friends... So, she [the psychologist] was very helpful about humanising how we are staff member should feel as well and how that that kind of stuff is going to impact on us.” (Physio, 25 years)

For others, the very fact they had taken a full day to focus and reflect on their practice, stepping away from the urgent requirement to perform, was a feature of the course they valued indicating reflective practice, as a core component of learning even when under high pressure.

### Changing practice, and reconceptualising roles

Participants reported changes in their clinical practice as a direct result of attending the course. This is in part due to an expansion of their rehabilitation tool kit noted previously. Several examples of using new tools were described as noted here:

“So, I’m using the sheet that I’ve made when they step down of ICU, it’s like something I use to develop a rapport with patients, which has been really useful, to have by the bedside and having an outcome measure that that the patient can see. And also, I can see which is helpful. …now I slightly feel like our cohort is much more rehab heavy, which is really nice. I’ve just today started doing an ICU diary for one of our patients, which I’m gonna go through with him tomorrow… But I definitely don’t think I would have even thought about that if I hadn’t gone on the course.” (Physio, 5 months)

The insights gleaned from the inter-professional presentations and discussion were also noted as having a direct impact on practice:

“the other day I saw a dietitian on the ward…I wanted to grab her quickly before she saw the patient and say that the patient couldn’t eat an apple because they didn’t have their dentures and they are weak and fatigued and I just wanted to make you aware. It was the most basic things that I learned on the course” (Physio, 1 year)

Participants further commented that the course impacted on wider aspects of professional development. Some described how their professional network had grown and others about initiating discussions with the wider MDT about developing new weaning teams. A number commented how they had shared the content and experience of the course with colleagues, some of whom had also attended. This helped to bring them together as a team, allowing them time to share their experiences and reflect on their rehabilitation sessions. This vital time to learn and reflect has enabled them to develop their practice going forward:

“We’ve also tried to make time as a team to come back and share our experiences of what we all took from it (the course)… we’ve given you the wider team chance to sit down and reflect as a team… which is really good and it’s good to…reflect as a team and look about moving forward what we would do differently next time… that was definitely, supported and through learning from the course.”(Physio, 7 years)

## Discussion

The findings from the CIRLC-rehab evaluation build on our findings from the CIRLC-acute module,^10^ both of which demonstrated how learning technologies can be used to create innovative solutions to education that optimises the skills of the available workforce and maintain social distancing. Both courses demonstrated an increase in candidate confidence before and after attendance; however, in addition to this, the mixed-methods evaluation reported in this article has shown direct changes to clinical care due to attending this course.

The IDT involvement both in relation to the delivery of content and participants themselves was an important factor – expanding candidates’ holistic view of care and demonstrating evidence of enhanced IDT working in practice. The reiteration in many accounts of the nutrition teaching indicates a gap in general awareness of this core aspect of rehabilitation – this is something that should be considered in other education courses in critical care.

The candidate experience was enhanced through the interactive tutorials and capacity to discuss and share experiences in a physically and psychologically safe environment. For many, this was the first opportunity to ‘take stock’ and a suitable forum for reflection on their practice, to develop new ideas and informal networks, and to discuss current UK practices which were sometimes inconsistent.

The tutorials also provided time for candidates to consider themselves within the pandemic as well as the patient. This space for personal reflection was an unexpected, but important finding and indicates a need to address consistent and regular access to psychologically safe places for reflection, in education and clinical practice, for all frontline workers and educators.

## Key lessons and implications for rehabilitation

1. This course format was a successful way of delivering standardised training in the knowledge of holistic rehabilitation in critical care. This could be adopted as a pedagogical approach more broadly in many disciplines and topic areas.
2. The MDT delivery of the course facilitated understanding of early rehabilitation strategies from the perspective of all disciplines. Furthermore, it was able to stretch IDT expertise and presence, which can be patchy in terms of certain disciplines, for example, psychology, across critical care settings.
3. Qualitative data suggested that understanding of nutrition is poor, and this should be something to improve in ongoing in critical care education.
4. Dehumanisation of both the patient and staff was exacerbated during the pandemic due to the environment and skill-based team approaches to care. Having a virtual space to reflect together, to re-centre on the patient and to recognise this as shared experience was an unexpected benefit of the course and needs to be considered in critical care training and service delivery more broadly. Training courses might also provide an important opportunity to highlight staff support offers for the critical care workforce.
5. Offering opportunity and roles for those who were unable to be on the frontline, yet held significant expertise, was an important use of skill and resource for the broader benefit of the critical care workforce.
